# Decoding the Bell-Shaped Calcium Spikes in Phosphorylation Cycles of Flagella

**DOI:** 10.3390/ijms23073760

**Published:** 2022-03-29

**Authors:** Miljko Satarić, Tomas Nemeš, Jack Tuszynski

**Affiliations:** 1 Faculty of Technical Sciences, University of Novi Sad, 21000 Novi Sad, Serbia; sataric.miljko@gmail.com (M.S.); nemes.tomas@uns.ac.rs (T.N.); 2 Serbian Academy of Sciences and Arts, 11000 Belgrade, Serbia; 3 Department of Physics, University of Alberta, Edmonton, AB T6G 2R3, Canada

**Keywords:** flagellum, microtubule, axoneme, dynein motors, calcium spike signaling

## Abstract

We investigate the messenger role of calcium ions implicated in the regulation of wave-like bending dynamics of flagella. The emphasis is on microtubules of flagellar axoneme serving as nonlinear transmission lines for bell-shaped spikes of calcium ions. The calcium sensitive proteins, such as calmodulin, exhibit activation dependence on the spike train frequency and amplitude. Here, we analyze a Ca^2+^ decoding module IDA-I1 whose activity is controlled by Ca^2+^ activated kinase. We find that trains of Ca^2+^ spikes are advantageous compared to a constant rise in Ca^2+^ concentration as being more efficient and much less prone to noisy fluctuations.

## 1. Introduction

Cilia and flagella are characterized by different patterns of movement but are identical in structure and composition. In the following, for the sake of brevity, we will mostly use the term flagella. Flagella are long thin appendages of many living cells, whose oscillatory bending waves enable cells to be propelled through visco-elastic fluids, or to drive fluid flows across the surface of the cell. Motile flagella are capable of complex, subtly coordinated movements and can play versatile roles in fertilization and embryonic developments [1]. Specific flagella perform the windshield wiper-like actions in trachea, where they clean mucus out of lungs. A flagellum of a sperm cell forms its “tail” and propels it in order to swim at speeds up to 3 mm/minute and attach to and fertilize an egg. The hallmark structure of flagella is their central core, the axoneme.

**Figure 1:**
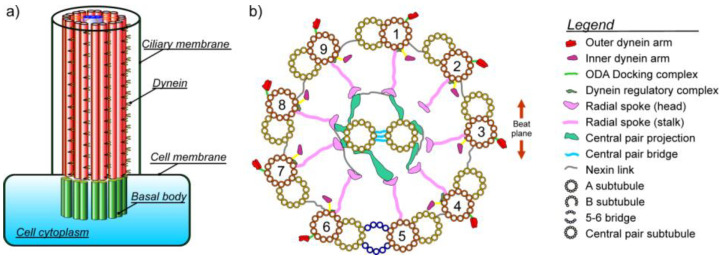
(**a**) Cylindrical architecture of the flagellum with nine microtubule doublets forming the axoneme; (**b**) cross section of axoneme. The figure shows the main ingredients; nine microtubule doublets, the central microtubule pair, radial spokes, nexin linkers, and two sets of different dynein motors (outer dynein arms and inner dynein arms). Some accessory proteins are also added [2].

The axoneme emanates from the flagellum cell body. The part inside the cell that anchors axoneme is called basal body, Figure 1a.

The microtubule (MT) cytoskeleton of flagella consists of triplet MTs in the basal body and microtubule doublets (MTDs) in the axoneme. The axoneme contains 9 MTDs and the central pair (CP) of single MTs, all in a parallel cylindrical arrangement. The MTDs and CP are connected by the radial spokes (RSs), while neighboring MTDs are linked with sets of nexin-linkers, see Figure 1b. The axonemal cytoskeleton is endowed with hundreds of accessory protein structures, all encapsulated in the flagellum’s plasma membrane [1,3].

More than 600 different proteins were detected from the *Chlamydomonas* axoneme by mass spectroscopy-based proteomics. Among these proteins just MTs and dynein motors play the executive roles for flagellar bending motions. These motions are driven by ATP-induced conformational changes of dynein motors. Other accessory proteins constitute a complex toolkit for subtle support and aid in flagellar dynamics. It is known that axonemes are reinforced by a network of tektin protofilaments that together with nexin-linkers maintain the nine-fold integrity of axoneme and enhance its mechanical robustness. Some accessory complexes are indicated in Figure 1b.

### 1.1. Microtubules

**Figure 2:**
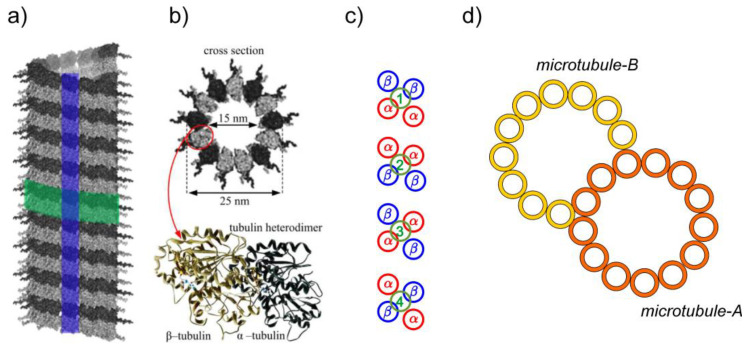
(**a**) MT cylinder of 13 parallel protofilaments (one protofilament is depicted in blue, helical symmetry in green; (**b**) the cross section of a MT with denoted dimensions, and tublin α-β hetero-dimer (secondary structure); (**c**) four types of nanopores in MT wall; (**d**) the microtubule doublet consists of A-MT and B-MT, with 13 and 10 protofilaments, respectively [2].

Microtubules are long hollow cylinders (Figure 2a) typically containing 13 parallel protofilaments of α-β tubulin heterodimers (Figure 2b). There exist four types of nano-pores in the MT wall located between neighboring tubulins connecting MT lumen with an external part of a MT, (Figure 2c). These nanopores are very important for calcium signaling along MTDs of axoneme as will be shown in the context of our model. The outer diameter of a MT is 25 nm. Every MTD is composed of one A-MT and one B-MT. The A-MT contains the usual number of 13 protofilaments and is tightly fused with B-MT composed of 10 protofilaments, see Figure 2d. MTs can be generally highly dynamic in the cytoskeleton, but in the case of the flagellar axoneme MTs are stable and have a constant length. They are parallel to each other with MTD inter-spacing of about 30 nm, which is closely comparable to the diameter of an MTD itself. Axonemal MTDs are implicated in very versatile flagellar functions; starting from the construction of the axoneme’s architecture and stability and playing the role of railways for intra-flagellar transport, then providing a complex mechanism for flagellar bending. The transport along the axoneme is essential for building and maintenance of eukaryotic flagella. Kinesin II motors carry cargoes in an anterograde mode, from the proximal to the distal end of the axoneme, using B-MTs as rails, while cytoplasmic dynein-1b (larger motors) carry cargoes in the opposite direction using A-MTs since these are more spacious paths [4]. These functions are perhaps the main reason why instead of single MTs the axoneme comprises MTDs. In most flagella the MTDs numbered 5 and 6 in the standard numbering convention are permanently linked to one another and cannot slide relative to each other. They do not have dynein motors attached to them. This fact defines the position of the beating plane of the axoneme, (Figure 1b).

### 1.2. Dyneins

Axonemal dyneins are structured in two complexes of inner and outer dynein arms (IDAs and ODAs—Figure 1b). There is a strikingly large difference in structure and organization between ODAs and IDAs as well as with regard to their function. The ODAs have two versions; one with a dimer (two dynein heavy chains-DHCs) and the other with a trimer (three DHCs). They also possess light chains (LCs) and intermediate chains (ICs). Species with dimers have protein calaxin as an associated Ca^2+^ sensor while trimers are associated with calmodulin (CaM) LC4 as a Ca^2+^ sensor. The ODAs provide much of the power required for flagellar movement. The ODAs repeat once every 24 nm and are very homogenous in the structure of the axoneme.

The IDAs are much more complex and contain at least 12 different DHCs [5,6]. These DHCs are arranged and organized into seven different IDAs, labeled (a, b, c, d, e, I1, and g) and are distinct in composition and respective location in a 96 nm repeating unit in the axoneme. The IDA complex is responsible for control of the size and shape of the forward and reverse flagellary bends. Special significance in this mechanism is assigned to the major IDA motor I1. It is located in the proximal end of each 96 nm axonemal repeat near the radial spoke (RS1) and it makes contacts with many other structures in the axoneme including the nearest ODA and neighboring single-headed IDAs. The I1 comprises two DHCs (1α and 1β), three intermediate chains (IC97, IC140, IC138), and five light chins. Importantly, the subcomplex IC138 is essential for the control of flagellary waveform in terms of Ca^2+^ signals. The phosphorylation is mediated by the modifier of inner arms (MIA) complex present in RS1. For example, MTD sliding in *Chlamydomonas* flagella is regulated by phosphorylation/dephosphorylation of the IC138 complex of I1-IDA. The phosphorylation and dephosphorylation of this protein inhibits and restores wild-type MTD sliding, respectively.

It should be strongly stressed here that effective functioning of I1-IDA is provided in terms of Ca^2+^ signaling. The I1 acts as a brake to slow and locally regulate MTDs sliding driven by ODAs and other IDAs. Its 1β-DHC contributes to force production while 1α-DHC is the motor domain that resists or limits MTD’s sliding. Below we elaborate on the decoding mechanism of calcium oscillations involved in the activation of I1 dyneins. This represents the main focus of this article.

### 1.3. Radial Spokes

The RSs act as spacers to position MTDs in a circle around a central pair of MTs. These RSs are large complexes comprising at least 23 different subunits including a diverse group of structural and signaling proteins. They represent two important regulatory hubs aimed at tuning of flagellar motility. The first spoke RS1 in association with I1 dynein is positioned near the proximal end of the 96 nm axonemal repeat [7]. The second spoke RS2 and associated CaM, along with the spoke-associated complex (CSC) and nexin-dynein regulatory complex (N-DRC) are located at the distal end of the 96 nm repeat. Together, these two hubs coordinate dynein activity and regulate the waveform and beat frequency of the flagellum. Below, we elucidate how calcium signaling pathways participate in this regulation.

### 1.4. Bending of Axoneme

The MTDs are not contractile but, instead, slide relative to one another under the action of forces and torques, produced by dynein motors. The variability of sliding velocity is the basis of flagellar bend formation. Very notably the different IDAs and ODAs undergo MTDs translocation at different speeds.

Bending of the axoneme originates from the imbalance of dynein motors on the opposite sides of the bending plane. For instance, switching of dynein activity between MTD7 and MTD3 (Figure 1b) is thought to be responsible for the periodical and planar oscillations of sperm flagella.

During their power stroke dyneins produce force that tends to slide attached MTDs with respect to each other, thus regulating the beat pattern of the axoneme. Accordingly, the flagellar beat is enabled by alternating episodes of activation of sets of dynein. It is concluded that these beats are highly organized processes such that Ca^2+^ signals indirectly activate dynein sliding, initiating the beat, and conversely, the beat tunes the dyneins. When activated, dyneins on one side of the axoneme win the tug-of-war, leading to relative motion between MTDs. Passive nexin-linkers and RSs, along with the basal body of axoneme, constrain sliding and convert it into bending [8].

## 2. Principles of Calcium Signaling

Calcium, being the most abundant metal on the earth and the fifth most abundant element in the human body, was adopted as a cellular regulator at an early evolutionary stage. Calcium acts as an intracellular second messenger in living processes ranging from cell movement, important here for secretion and contraction, up to gene expression and even cell apoptosis. The ability of a simple ion such as Ca^2+^ to play a pivotal role in cell biology results from the ease with which cells have to shape Ca^2+^ signals in the dimensions of space, time, amplitude, and frequency [9,10].

The advantage of calcium is its specific coordination chemistry. This is determined by its valence, the ionic radius of 0.99 Å and its radius of hydration of 4.5 Å. A Ca^2+^ ion can accumulate 8–12 oxygen atoms in the primary coordination sphere. The range of Ca-O distances in complexes is 0.23–0.28 nm. The abilities of Ca^2+^ and negative phosphate ions to trigger changes in the shape and charge of different proteins are two universal tools of cellular signaling transduction. Thus, Ca^2+^ binds to thousands of different proteins with over a million-fold range of affinities (nM to mM). These binding events can influence protein’s change in localization, association, and function. Interestingly, cells spend a significant part of their totally consumed energy to maintain changes in Ca^2+^ concentration keeping the difference between their intracellular (0.1 μM free) and extracellular (mM) concentration [11].

In extracellular fluids, the concentration of Ca^2+^ varies between 2.0 and 2.6 mM, subdivided into three components: (a) ionized, (b) bound to small inorganic molecules, and (c) complexed with organic molecules. The concentration of ionized Ca^2+^ forms is of the order 1 mM. The total Ca^2+^ concentration in the cytosol is also on the order of mM. But there, the concentration of free ionized fraction is about 10^4^-fold lower than the bound and complexed components. To this end, cells are endowed with specific organelles which contain sites that ligate Ca^2+^ with significant affinities. The first group of such ligand peptides are sequestered inside mitochondria, the endoplasmic reticulum and the sarcoplasmic reticulum (ER; SR), as well as in the Golgi apparatus. The second class of such proteins involves cytoskeletal structures: microtubules, actin filaments, and intermediate filaments. All these organelles buffer free Ca^2+^ within the nM range without modifying its total content in the cell. A very important fact for this article is that ERs and SRs are missing in mature flagella, including sperm cells. However, there are the ER remnants in the membranous forms with similar Ca^2+^ buffering properties to those of the ER.

It is known that axonemes in mammalian sperm cells are reinforced by a network of tectin-dense fiber axoneme complexes that together with nexin-linkers maintain the nine-fold geometry and enhance the mechanical robustness of the axoneme. A significant role in controlling Ca^2+^ concentration in sperm cells flagella is played by mitochondria. Besides energy homeostasis, mitochondria play a vital role in maintaining ion homeostasis in sperm cells [12].

**Figure 3:**
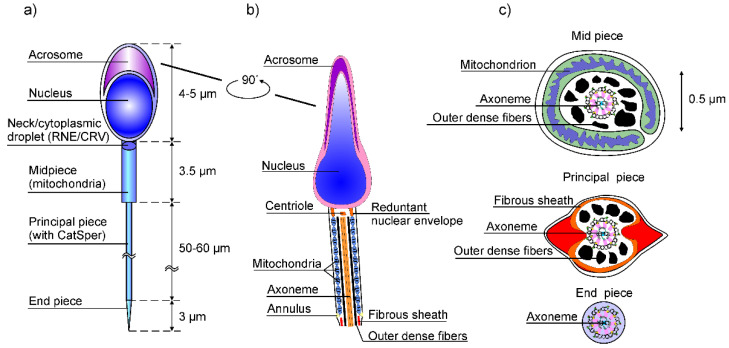
(**a**) Graphical illustration of the human sperm cell with its segmentation, indicating the spatial dimensions; (**b**) the cell head and mid-piece, with some details; and (**c**) the cross-sections of the mid-piece, principal-piece, and end-piece.

Figure 3 illustrates the organization of the human sperm cell with its segmentation, indicating the spatial dimensions. The mitochondria are restricted to the mid-piece of the flagellum. They wrap helically around the outer dense fiber axoneme complex during spermatogenesis to form a cylinder-shaped mitochondrial sheet coaxial with the axoneme [13].

Within the cylinder-shaped sheet, adjacent mitochondria associate both end-to-end and along their lateral surfaces. This arrangement sets a concentrated array of mitochondria closely to the flagellum membrane from outer side, Figure 2c. This arrangement offers an efficient way to provide most of the ATP energy supply required for flagellar motility. Additionally, the vicinity of the axoneme and Ca^2+^ ionic channels enables mitochondria to support the calcium induced calcium release (CICR) process which underlies the pulsatile localized calcium waves along the flagella. The mammalian sperm mid-piece contains 50–57 mitochondria with diameters in the 0.7–3 μm range, which represents about 10% of the cell volume. It was demonstrated in [14] that Ca^2+^ wave activities in Xenopus leaves oocytes strengthened by oxidizable substrates that energize mitochondria, thus increasing the Ca^2+^ wave amplitude and velocity. This clearly describes the basic role of mitochondria in intracellular Ca^2+^ signaling. Therefore, mitochondria may be safely considered as the guardians of the gate between life and death since they also play an important role in cell apoptosis.

There is also clear evidence that other Ca^2+^ storage organelles are contained in mature mammalian sperm cells due to the presence of inosital triphosphate receptors (IP3Rs) in the acrosomal part of the cell head and over the sperm neck and mid-piece of the flagellum.

## 3. Calcium Signaling in Flagella

Flagella change their motility in response to Ca^2+^ concentration which is a crucial regulator of the modulation of flagellar movement. These modulations include: (1) changes in flagellar waveforms; (2) reversal of the direction of flagellar bending; (3) arrest of bending; (4) changes in the beat frequency and amplitude [15].

For successful Ca^2+^ signaling there should exist four functional segments as follows:

1. In the case of the human sperm, the signaling is triggered by a stimulus (progesterone or nitrogen monoxide -NO) that generates Ca^2+^ signal through Cat-sper channels;
2. It activates the ON mechanism that feeds Ca^2+^ into flagellum and sperm head. Since flagella are thin cylinders with a very large surface-to-volume ratio [16], these Ca^2+^ fluxes are efficiently injected into the cytosol.
3. The pulses of Ca^2+^ ions function as messengers, which stimulate several axonemal proteins, primarily CaM, calaxin and IC138 of IDA-I1, to perform the control of dyneins and to modulate flagellary beats [17].
4. Eventually the OFF mechanism, composed of pumps and ionic exchangers, removes Ca^2+^ from the cytoplasm to internal Ca^2+^ stores and buffers, as well as out of flagella, in order to restore the resting state [18].

When activated, both Ca^2+^ entry and release channels introduce Ca^2+^ into flagellar cytoplasm. However, since these channels (for example Cat Sper) have short open times, they only introduce brief pulses of Ca^2+^ that form a small puff around the mouth of each channel [19,20].

These elementary Ca^2+^ signals are the building-blocks by which recruitment of the complex Ca^2+^ signals is constructed. This includes the localized “Ca^2+^ clouds” drifting along MTDs within the scope of our model [2,16,21].

The advantages of such pulse-like oscillating Ca^2+^ waves instead of global increases of Ca^2+^ concentration in controlling flagellary movement are manifold. These signals can have a rapid action considered to have a much higher fidelity of information transfer than simple tonic changes in Ca^2+^ concentration since they are much less prone to noisy fluctuations.

## 4. The Principal Sensors for Ca^2+^ Signals in Flagella

The main sensor for calcium signaling is a very ubiquitous Ca^2+^ binding protein CaM. It bears structural Ca^2+^-binding motifs known as “EF-hands”. The affinity of Ca^2+^ for CaM is $K_{d}\approx 1\;\mu M$ making it an ideal receiver for the rapid transient Ca^2+^ increase seen with each incoming localized spike. One of the best-known enzymes that uses CaM to help it “count” Ca^2+^ spikes is CaM dependent protein kinase C, which activates dynein motors ODAs and IDAs via phosphorylation [22]. As a corroboration of the essential role of CaM in calcium signaling, one can refer to the experiment in which a CaM antagonist W-7 inhibits flagellum motility initiation [23]. In sea urchins, *Chlamydomonas* and teleost fish sperm CaM affects the symmetry of flagellar beating.

Protein kinase C (PKC), which is activated via diacyglycerol in the presence of Ca^2+^ is important for motility maintenance via phosphorylation of flagellar proteins involved in dynein complexes [22,24].

The second important Ca^2+^ sensor is neuronal calaxin, which directly acts on an ODA and regulates specific flagellar movement during sperm chemotaxis. Calaxin is essential for generation and propagation of Ca^2+^-induced asymmetric flagellar bending [17,25]. The formation and propagation of asymmetric beating waves in sperm flagella depend on Ca^2+^ concentration, which regulates calaxin activity in catalyzing ODAs speeds. Immunoelectron microscopy and biochemical analysis showed that calaxin interacts with the ODA heavy chain in a Ca^2+^-dependent manner. Knockout of calaxin caused reduced motility in sperm flagella. In some cases, ODAs on the doublets 3, 5, and 8 were lost, suggesting that calaxin might play a role in stabilizing the association of ODAs with MTDs within axoneme.

## 5. Mechanism for Propagation of Intracellular Calcium Waves—The Particular Aspect of Flagellar Waves

Calcium waves were first seen at the calcium tsunami, which crosses a fertilizing medaka fish egg [26]. These waves were subsequently inferred to cross a wide variety of cell parts, whole cells, and tissues with speeds ranging from $1\;\frac{\mathrm{nm}}{s}$ up to $3\;\frac{\mathrm{cm}}{s}$. Jaffe [27] presented collated evidence about different classes of signaling calcium waves in the context of various cellular processes. The long-standing dogma about the propagation of calcium waves in living cells relies on the autocatalytic mechanism called CICR. This is a regenerative cycle in which locally elevated intracellular calcium concentration induces the release of new Ca^2+^ ions stored in ER or SR organelles. These additional Ca^2+^ ions diffuse to nearby ER channels where they cause the release of yet more calcium. This self-sustaining cycle is primarily modulated by the increased levels of inositol (1, 4, 5) triphosphate (IP_3_) within the cytosol which forms calcium channels by the receptor proteins IP_3_Rs. These intracellular channels respond to Ca^2+^ concentration in a bell-shaped fashion; that is IP_3_R is inactive at low nM concentration of Ca^2+^ and active at mid-μM concentration, then inactivated again by high concentrations that are in the mM range of Ca^2+^. Thus, the speed of CICR waves is determined by the diffusion of Ca^2+^ ions combined with diffusion of IP_3_Rs. The diffusion constant of Ca^2+^ ions is of the order of $20\;\frac{{\mu m}^{2}}{s}$ while for IP_3_Rs is much greater, namely $300\;\frac{{\mu m}^{2}}{s}$ [28].

If the density of IP_3_Rs is higher, the speed of Ca^2+^ waves is further increased. We already stressed that ER and SR are missing in mature flagella. However, the presence of IP_3_Rs in the sperm cell’s head, neck, and mid piece (Figure 3) indicates that this CICR mechanism can be achieved in these compartments, especially in mid piece, where the mitochondrial sheet around the axoneme is expressed.

The activation and inactivation of IP_3_Rs within CICR mechanism occurs on a faster time scale than the production and degradation of IP_3_. The Ca^2+^ activation and inactivation of IP_3_Rs manifests itself on the time scale of milliseconds, while the production and degradation of IP_3_ follows the time scale of several seconds.

The characteristic speeds of Ca^2+^ waves enabled by the CICR mechanism are of the order of 3–35 $\frac{\mu M}{s}$ [13,27]. For example, the speed of waves in Guinea pig myocytes is $32\;\frac{\mu m}{s}$ [29]. However, of greater interest for our concept presented here is the contribution by Jaffe [30] regarding fast calcium-induced calcium influx (CICI) waves. Much of that model concerns calcium waves along functional flagella, including sperm cells and their principal piece. These waves have the speeds ranging from 10^2^ to 10^3^ $\frac{\mu m}{s}$ in a wide variety of systems. Huang et al. [31] observed intracellular calcium waves in human fibrosarcoma cells with a speed of 100 $\frac{\mu m}{s}$. These waves were blocked by the calcium channel blockers, ions of gadolinium. Additionally, the speed of Ca^2+^ waves in human sperm was earlier estimated to be of the order of 500 $\frac{\mu m}{s}$ [32] and in the case of Hamster sperm (activated) was about 700 $\frac{\mu m}{s}$ [33].

We earlier established [21,34] and further refined [2,16] the polyelectrolyte concept of MTs aimed to explain how Ca^2+^ ions, albeit present in nM to μM concentrations in the cytosol, use MTs as a buffers to condense close around their filaments and C-termini (CTTs). Moreover, the accumulated Ca^2+^ ions form bell-shaped “Ca^2+^ clouds” that propagate along MTs much faster than it could be achieved by simple three-dimensional diffusion. The estimated speeds of Ca^2+^ waves within the scope of our model compared with some prior experimental results are given in Table 1.

The differences within our model are the consequences of different estimates of capacitance and resistance of elementary electric building blocks of MTD viewed as nonlinear electric transmission line. These differences are caused by different post-translational modifications of CTTs which change the capacitance of these building blocks. Nonetheless, fairly satisfactory agreement of our results with experimental data indicates that our concept offers a very promising mechanism capable to explaining an efficient signaling pathway of Ca^2+^ ions implicated in the modulation of flagellar bending.

## 6. Decoding of Axonemal “Ca^2+^ Clouds” by Phosphorylation Cycles

Prior experimental evidence has demonstrated that signaling by intracellular Ca^2+^ repetitive spikes regulates sensitive proteins in a way such that strengthening of appropriate stimulus increases spikes frequency [35]. For example, the extracellular stimulus speract induces changes of intra-flagellar Ca^2+^ concentration with oscillatory spatio-temporal evolution from the flagellum to the sperm head. In the presence of 0.5 nM speract as agonist, the frequency of Ca^2+^ pulses in Sea urchin sperm is of the order of 1 Hz, while increased agonist concentration of 0.1 μM generates a frequency of about 5 Hz [36]. Signal information can also be encoded in the amplitude of Ca^2+^ pulsatile train. This amplitude usually can be changed with the external stimulus [37]. Experimental data additionally indicate the capability of CaM and protein kinases to decode the amplitude of Ca^2+^ pulsatile signal into an appropriate cellular response [38]. Furthermore, a very important catalyzer of flagellar movement, CaM-dependent kinase C is sensitive to the frequency of Ca^2+^ signals [22,39]. It appears that such Ca^2+^ repetitive signals can be more efficient for protein phosphorylation if compared to constant Ca^2+^ signals of equal average concentration.

Here, we consider the activation-inactivation cycle of an axonemal target motor protein, for example an IDA-I1 which plays the fundamental role in the regulation of MTDs sliding. Its activation is triggered by phosphorylation through Ca^2+^/CaM-dependent protein kinase II (PKII), and it is inactivated by dephosphorylation [40]. The stimulation of the associated PKII is enabled by the binding of *n* Ca^2+^ ions to the E-F hands domains of the associated CaM molecule. Thus, the Ca^2+^/CaM-dependent PKII exerts the phosphorylation of IC138, the vital ingredient of the IDA-I1 motor protein. This can be considered a rapid “post-translational” modification accompanied by the conformation of target proteins. As an example, the phosphorylation and dephosphorylation of IC138 inhibits and restores wild-type MTDs sliding, respectively in *Chlamydomonas* flagella.

The fraction of activated PKII by Ca^2+^/CaM, denoted by Y(t), and the phosphorylated target protein IC138, denoted by X(t), obey the following time-dependent evolution equations:

(1) $$\frac{dY}{dt}=a_{Y}{\left[C\left(t\right)\right]}^{n}\left(1-Y\right)-b_{Y}Y$$

(2) $$\frac{dX}{dt}=a_{X}Y_{T}Y\left(1-X\right)-b_{X}X$$

Here, $Y_{T}$ represents the total available concentration of PKII in the axoneme, while the time course of the Ca^2+^ concentration within a single spike is denoted by C(t). Symbols $a_{Y}$ and $b_{Y}$ are the rate constants of Ca^2+^ binding and release in CaM, respectively, while $a_{X}$ and $b_{X}$ represent the rate of phosphorylation and dephosphorylation of IC138 by PKII, respectively. This is basically responsible for IDA-I1 motor actions in terms of the respective two heavy chains (1α, 1β).

Next, we implement our concept of bell-shaped spikes, so called “Ca^2+^ clouds” propagating along microtubule doublets of the flagellar axoneme. The scenario of such trains of spikes is elaborated in [16,21]. Similar models are developed in [41]. Interestingly our concept is widely elaborated from a mathematical perspective [42,43,44].

**Figure 4:**
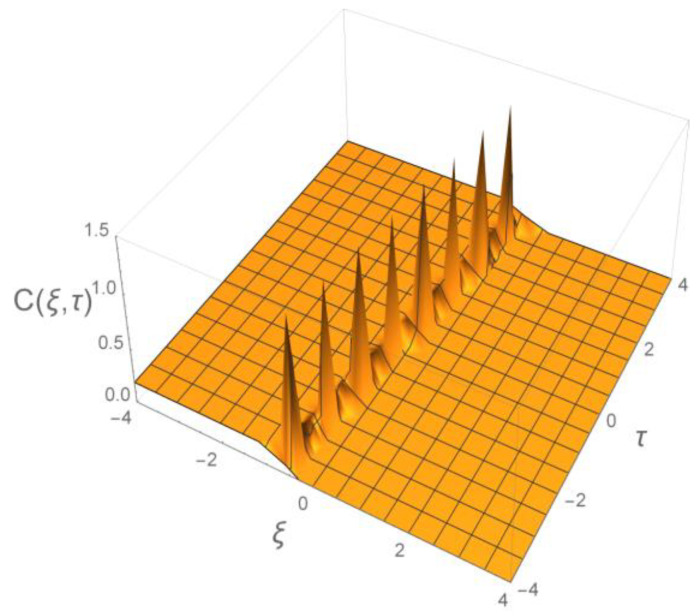
Our 3D representation of the train of spikes according to model described in Zahran [42]. Dimensionless space-time variables are: $=\frac{x}{\ell }$; $\tau =\frac{vt}{\ell }$.

The train of spikes arising from our model obtained in [42] is presented in Figure 4.

Equation (1) can be solved if the local Ca^2+^ concentration within a single spike is taken to be our bell-shaped “Ca^2+^ cloud”, [2,16]:

(3) $$C\left(x,t\right)=\frac{C_{0}}{{\left(cosh\left(\frac{x}{\ell }-\frac{vt}{\ell }\right)\right)}^{2}}$$

where x is the direction of an MT doublet, ℓ=8 nm is the tubulin dimer length, v is the drift velocity of “Ca^2+^ cloud”, *t* stands for the time variable, and $C_{0}$ is the amplitude of Ca^2+^ concentration within each spike. We should fix the position of a specific dynein motor with accompanying regulatory proteins (CaM, CaMKII, IC138) involved in the motor’s activations. Hence, we can take x=0 for this site and the time evolution of the local Ca^2+^ concentration now reads

(4) $$C\left(t\right)=\frac{C_{0}}{{\left(cosh\left(\frac{vt}{\ell }\right)\right)}^{2}}$$

Then, going over to the dimensionless time in Equation (1) by substituting

(5) $$\tau =b_{Y}t$$

and inserting Equation (5) into Equation (1) we obtain

(6) $$\frac{dY}{d\tau }+\left[{\left(\frac{C_{0}}{\kappa {\left(cosh\left(\frac{vt}{\ell b_{Y}}\right)\right)}^{2}}\right)}^{n}+1\right]Y={\left(\frac{C_{0}}{\kappa {\left(cosh\left(\frac{vt}{\ell b_{Y}}\right)\right)}^{2}}\right)}^{n};\;\kappa ={\left(\frac{b_{Y}}{a_{Y}}\right)}^{\frac{1}{n}}.$$

The parameter κ represents the half saturation concentration for Ca^2+^/CaM activated PKII. The bound number of Ca^2+^ ions in CaM (a so-called Hill coefficient) is n=4.

We can solve Equation (6) numerically for an increasing set of parameter values ε which is the ratio of the Ca^2+^ peak concentration and PKII half saturation

(7) $$\varepsilon =\frac{C_{0}}{\kappa }=\left\{1;1.5;2.0;,5.0\right\}.$$

**Figure 5:**
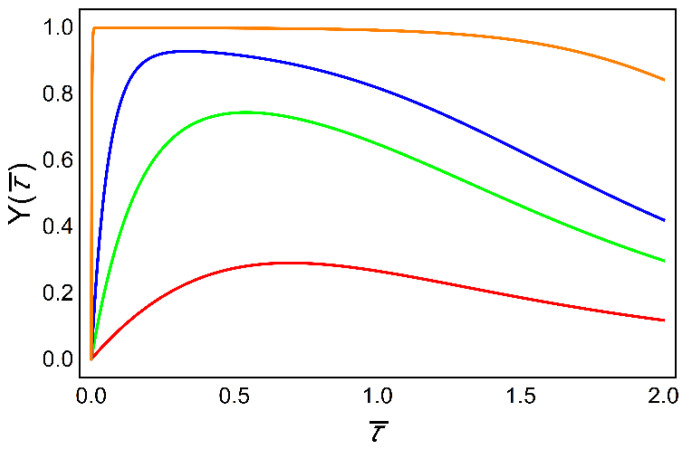
Calcium/calmodulin activation and inactivation increases with increasing amplitude of “Ca^2+^ cloud spikes” (ε=1 red, ε=1.5 green, ε=2 blue, ε=5 orange). The effective dimensionless time is now $\bar{\tau }=\frac{vt}{\ell b_{Y}}$.

The initial condition is: Y=0 for t=0. The shapes of resultant functions of $Y\left(\bar{\tau }\right)$ are depicted in Figure 5.

In flagella, it is quite reasonable to assume that Ca^2+^ binding to CaM is much faster than it’s unbinding ($a_{Y}\gg b_{Y})$ [45]. It implies that the ratio ε is several times greater than unity for spikes with amplitudes high enough. This is reflected in the above curve with ε=5 (see Figure 5, orange line), which is practically flat, giving

(8) $$\frac{dY}{d\bar{\tau }}=0,$$

equilibrium for activated PKIIs.

This implies the following solution of Equation (1):

(9) $$Y=\frac{1}{1+\frac{b_{Y}}{a_{Y}}{\left[{\left(cosh\left(\frac{vt}{\ell b_{Y}}\right)\right)}^{2}\right]}^{n}};\;\frac{b_{Y}}{a_{Y}}\ll 1$$

This expression shows that Y does depend on time and for large t tends to be zero. But for t→0, such that the second term in the denominator of Equation (9) is much less than unity, this indicates that Y has a “constant” value approximately equal to unity as was in Figure 5, for ε=5. If a Ca^2+^ pulse has a greater speed, the segment corresponding to a “constant” Y is shorter in time, which is plausible.

Thus, by inserting Y(t) from Equation (1) into Equation (2), under the condition of Equation (8), yields the following form of the evolution equation for X:

(10) $$\frac{dX}{dt}=A_{X}\left(C\right)-\left[A_{X}\left(C\right)+b_{X}\right]X.$$

The effective phosphorylation rate of IC138 is the function of Ca^2+^ concentration within the spike progressing along the axoneme as follows:

(11) $$A_{X}\left(C\right)=a_{X}Y_{T}\left[\frac{1}{1+{\left(\frac{\kappa }{C\left(t\right)}\right)}^{n}}\right].$$

Again, we use here the spike as the bell-shaped “Ca^2+^ cloud”, Equation (3), and insert it into Equations (10) and (11), yielding

(12) $$\frac{dX}{dt}=\left[\frac{a_{X}Y_{T}}{1+{\left(\frac{\kappa }{C_{0}}{\left(cosh\left(\frac{vt}{\ell }\right)\right)}^{2}\right)}^{n}}\right]-\left[\frac{a_{X}Y_{T}}{1+{\left(\frac{\kappa }{C_{0}}{\left(cosh\left(\frac{vt}{\ell }\right)\right)}^{2}\right)}^{n}}+b_{X}\right]X.$$

Introducing the new scaled dimensionless time into Equation (12) by substitution

(13) $$\tau =a_{X}Y_{T}t\;;\;dt=\frac{d\tau }{a_{X}Y_{T}},$$

one finds that

(14) $$\frac{dX}{d\tau }+\left[\frac{1}{1+{\left(\frac{\kappa }{C_{0}}{\left(cosh\left(\frac{v\tau }{\ell a_{X}Y_{T}}\right)\right)}^{2}\right)}^{n}}+g_{X}\right]X\left(\tau \right)=\left[\frac{1}{1+{\left(\frac{\kappa }{C_{0}}{\left(cosh\left(\frac{v\tau }{\ell a_{X}Y_{T}}\right)\right)}^{2}\right)}^{n}}\right],$$

where the dimensionless activation rate of the IC138 target is

(15) $$g_{X}=\frac{b_{X}}{a_{X}Y_{T}}.$$

In order to obtain numerical solutions of Equation (14) we should choose a set of appropriate parameters taking into account that the activation rate of the phosphorylation of IC138 $a_{X}$ is greater than its dephosphorylation rates $b_{X}$, as follows

(16) $$g_{X}=\left\{1;0.5;0.2;0.01\right\}.$$

**Figure 6:**
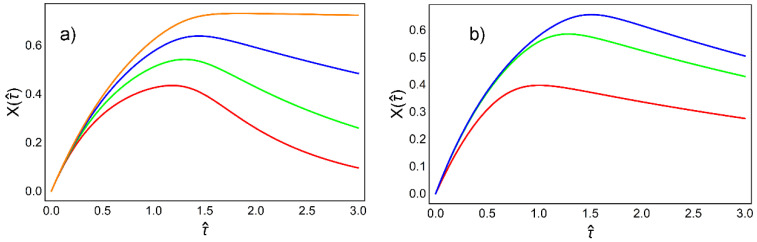
The target activation as a function of time. Increase of the spike amplitude along with decrease of dephosphorylation rate $b_{X}$ leads to an elevation of the activation curve for the target protein. Solutions of Equation (14) for: (**a**) different values of the parameter $g_{X}$ (1—red; 0.5—green; 0.2—blue; 0.01—orange) and fixed values of the κ and $C_{0}$; (**b**) different values of the ratio $\frac{\kappa }{C_{0}}$ ($\frac{1}{1.5}$ —red; $\frac{1}{3}$ —green; $\frac{1}{5}$ —blue) and fixed value of the dimensionless activation rate $g_{X}=0.2$. The effective dimensionless time here is $\hat{\tau }=\frac{v\tau }{\ell a_{X}Y_{T}}$.

We also adopt the reasonable physiological values of concentrations given as: κ=(0.3−0.4) μM and $C_{0}=1.5\;\mu M$. The Hill coefficient is n=4 again. Finally, the initial condition for the solution of Equation (14) is: X=0 for τ=0. The corresponding graphical solutions for target activation X(τ) are represented in Figure 6.

Generally, Ca^2+^ activation of the target protein LC138 has a rapidly rising phase reached by a peak spike of Ca^2+^ concentrations of the order of 1 μM before slowly decaying when the spike drops to the basal Ca^2+^ concentration level. We stress that elevated global cytoplasmic Ca^2+^ concentrations can be very toxic so, instead of a sustained manner, Ca^2+^ signals in flagellum are commonly generated in terms of pulsatile forms. Since the spikes of bell-shaped “Ca^2+^ clouds” (Equation (3)) drifting along the axoneme are fast compared to the rate of phosphorylation of the target IC138 protein, it is reasonable to conclude that the cumulative effect of several spikes is responsible for an efficient activation of the I1-IDA motor.

**Figure 7:**
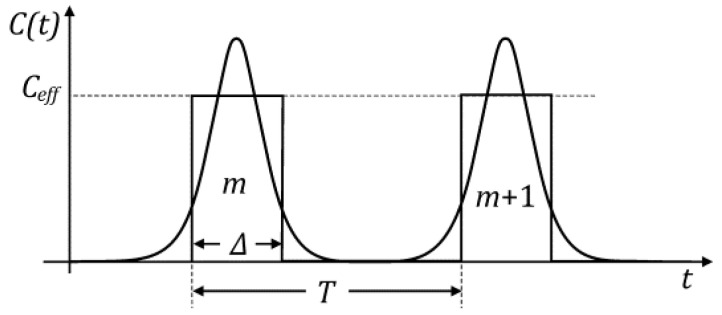
The approximation of a bell-shaped spike by a piece-wise constant function.

In order to analyze the cumulative activation outcome of a series of “Ca^2+^ clouds”, Equation (14) needs to be solved analytically but superposition of these spikes is analytically intractable. Therefore, we consider it semi-quantitatively by approximating the “Ca^2+^ clouds” with a piece-wise constant function having the equal profile area

(17) $$\int_{-\infty }^{+\infty }\frac{C_{0}}{{\left(cosh\tau \right)}^{2}}d\tau =C_{eff}\tilde{\Delta }=2C_{0},$$

where $\tilde{\Delta }$ is the dimensionless time width of a rectangular constant function in Figure 7. Taking $\tilde{\Delta }=2$ one gets $C_{eff}=C_{0}$, or for $\tilde{\Delta }=1$ it follows that $C_{eff}=2C_{0}$. In real time Δ is the time width and T is the spike train period (see Figure 7). The expression for that piece-wise function is given by:

(18) $$C\left(t\right)=\{\begin{array}{c} C_{eff},\;mT\leq t<mt+\Delta \\ 0,\;mT+\Delta <t<\left(m+1\right)T \end{array}\;m=1,2,3\ldots$$

The corresponding duty ratio is

(19) $$\gamma =\frac{\Delta }{T}.$$

We assume that the target protein IC138, in order to be phosphorylated, integrates the incoming spikes of “Ca^2+^ clouds”. In that respect, the average IC138 activity for a single m-th spike has the form

(20) $$\langle X_{m}\rangle =\frac{1}{T}\int_{0}^{\Delta }X\left(t\right)dt.$$

If we consider a spike train with T=1s and γ=1/2, then Equation (20) in terms of Equation (14) for $g_{x}=0.2$ yields

(21) $$\langle X_{m}\rangle =\frac{1}{T}\int_{0}^{1/2}X\left(t\right)dt=0.086.$$

We now use the analytical result for cumulative activation of the target protein for the spike train containing the greater number of piecewise functions of Equation (18) evaluated by [45] as:

(22) $$\langle \sum_{m=1}^{\infty }X_{m}\rangle =\langle X\rangle =\frac{\sigma }{\sigma +1}\left\{\gamma +\left(\frac{\omega \sigma }{\sigma +1}\right)\frac{\left[1-exp\left(\frac{-\gamma \left(1+\sigma \right)}{\omega }\right)\right]\left[1-exp\left(\frac{\gamma -1}{\omega }\right)\right]}{\left[1-exp\left(-\frac{1+\gamma \sigma }{\omega }\right)\right]}\right\},$$

where in our case the corresponding parameters, the effective activation, and duty ratio are given by

(23) $$\sigma =\left(\frac{1}{g_{x}}\right)\left[\frac{1}{1+{\left(\frac{\kappa }{C_{eff}}\right)}^{n}}\right];\;\gamma =\frac{\Delta }{T}\;.$$

The dimensionless frequency ω is the ratio of the frequency of Ca^2+^ spike repetition $\frac{1}{T}$ and the frequency of the dephosphorylation cycle ($\frac{1}{T_{dph}}=b_{x}$):

(24) $$\omega =\frac{1}{Tb_{x}}.$$

**Figure 8:**
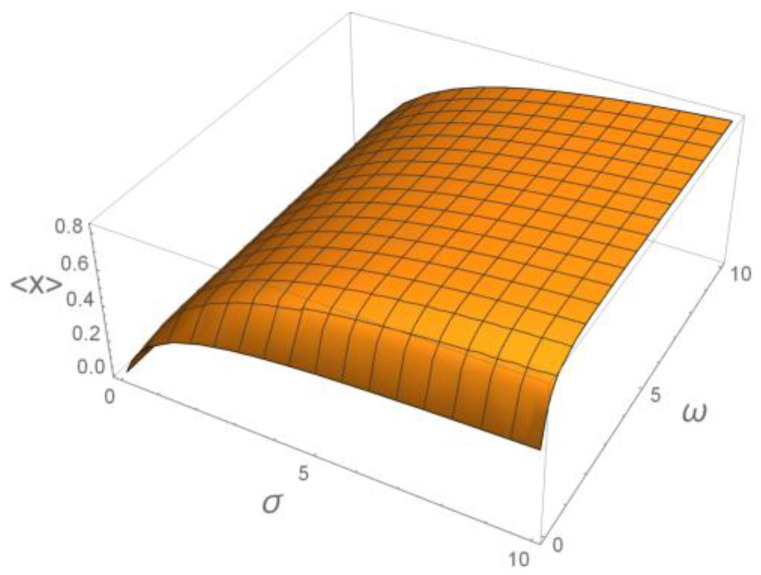
The 3D plateau-like function of cumulative activation of the target protein.

Now, we fix the duty ratio to be $\gamma =\frac{1}{2}$ and consider the function from Equation (22)

(25) $$\langle X\rangle =f\left(\gamma =\frac{1}{2};\sigma ,\omega \right),$$

representing it in the 3D form as shown in Figure 8.

As a special example, we can apply this concept to the flagellar Ca^2+^ spike trains from experimental assays with sea urchin sperm [46,47,48].

The estimated parameter values in this case are as follows:

(26) $$\gamma =\frac{\Delta }{T}=1/2;\;T=0.5s;\;b_{x}=0.2s^{-1};\;\omega =10;\;\sigma =5\;.$$

**Figure 9:**
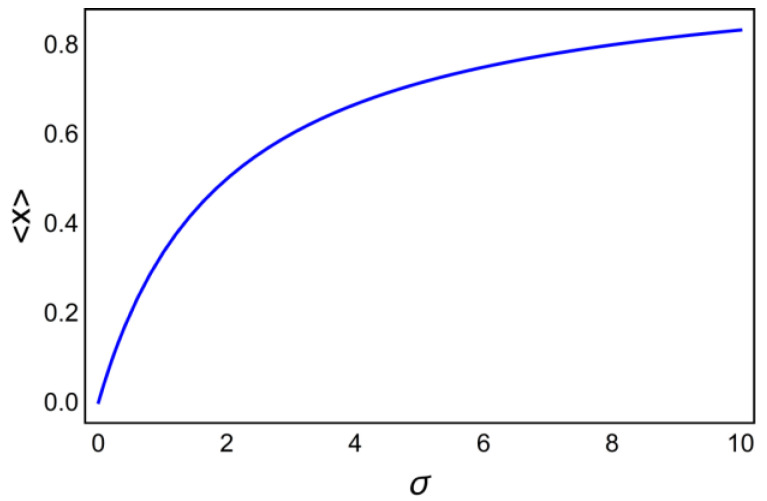
The cumulative activation as the function of the effective activation rate σ.

If we fix the frequency ω=10 and change σ we obtain from Equation (22) the function

(27) $$\langle X\rangle =f_{1}\left(\sigma ;\omega =10\right),$$

which is shown in Figure 9. It is clear that it saturates with increasing concentration of PKII and with increasing the phosphorylation rate $a_{x}$.

**Figure 10:**
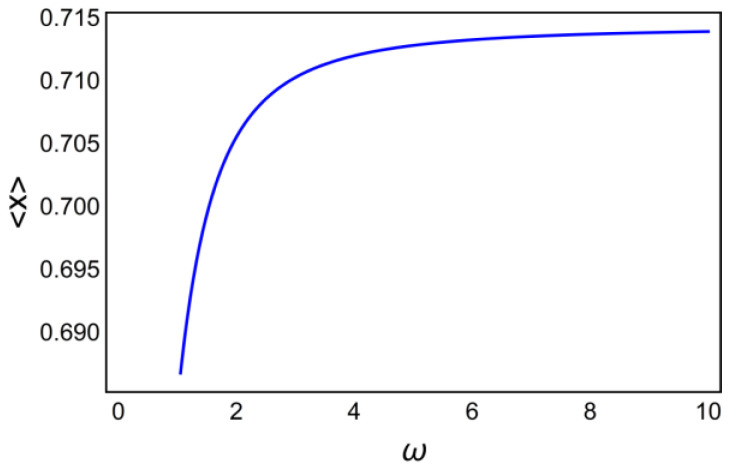
The cumulative activation as a function of the relative spike frequency ω.

Otherwise, setting σ=5, the cumulative activation as the function of frequency is

(28) $$\langle X\rangle =f_{2}\left(\omega ;\sigma =5\right)$$

and is presented in Figure 10.

The comparison of Figure 9 and Figure 10 shows that the frequency of spikes causes a steeper increase in the activation of the target protein, saturating at a value of ω≥10.

Eventually, on the basis of Equation (22) the cumulative activation of the LC138 complex in sea urchin sperm reads

(29) $$\langle X\rangle =f\left(\gamma =\frac{1}{2};\sigma =5;\omega =10\right)=0.714$$

On the basis of Equation (21) for the activation by a single bell-shaped spike, and using result of Equation (29) we estimate that approximately eight spikes are needed to achieve cumulative activation of the LC138 complex, a vital ingredient of the I1 inner dynein arm.

## 7. Conclusions

In this paper we provided a description of the structure and bending dynamics of flagella. Special emphasis was placed on the microtubules within the axoneme and the associated dynein motor proteins. We revisited the accumulated knowledge about 15 different axonemal dyneins that are distinct in both their composition and location within the axoneme. These dyneins are implicated in complex mechanisms of flagellar beating.

Specific attention has been dedicated to the unique signaling role of Ca^2+^ ions in the regulation of flagellar dynamics. For example, in sperm cells the intracellular Ca^2+^ level, pH, and ATP are the key regulatory elements of bending motility. The first two factors are controlled by ion channels. The principal Ca^2+^ channel is CatSper, which is activated by progesterone and flagellar alkalinity. The most important Ca^2+^ sensors in flagella are EF-hand proteins, primarily calmodulin and neuronal calaxin whose roles in Ca^2+^ signaling are very crucial.

We have paid significant attention to the concept of the bell-shaped “Ca^2+^ cloud”. Calcium ions injected into the flagellum by ionic channels (CatSper, for example), through a so-called calcium induced calcium influx, and further elevated by CICR release mechanism, accumulate closely around microtubules in the axoneme. These “Ca^2+^ clouds” are not static but instead, move with the speeds of the order of hundreds of micrometers per second, see Table 1. They form trains of narrow spikes, see Figure 4. Increased intracellular Ca^2+^ influx elevates their amplitude and speed. Such oscillations of concentration of free intracellular Ca^2+^ are a basic control mechanism for beating of flagella.

The main part of this article, which is our original and novel contribution, elucidates the decoding of these spike trains in the context of the phosphorylation cycle mediated by calmodulin and protein kinase. It appears that the decoding process is dependent on the amplitude and the frequency of these spikes within the train. We investigated the case where these spikes activate the target protein IC138 more efficiently if the frequency is higher. In that respect the oscillation frequency should be greater than the inactivation (dephosphorylation) rate of the IC138 target. As an illustrative example, we applied the concept of cumulative activation of IC138 phosphorylation to the case of Ca^2+^ signaling in sea urchin sperm. The obtained numerical estimation of dynein I1 activation has an important physiological relevance as demonstrated in Equation (29). Our analysis indicates that trains of Ca^2+^ spikes are advantageous compared to a constant rise in Ca^2+^ concentration as being more efficient and much less prone to noisy fluctuations. We expect that our results shed more light for deeper understanding of subtle calcium control mechanisms implicated in flagellar dynamics.

## Figures and Tables

**Table 1:** A comparison between our estimated speeds and experimental values measured by different authors.

Our Model (Satarić et al.)	Estimated Speeds [μm/s]	Experimental Evidence	Experimental Speed [μm/s]
2009 [34]	6000-overestimated	/	/
2010 [21]	160–240	Huang et al. [31]	100
2019 [16]	530	Mortimer et al. [32]	500
2020 [2]	620	Ishijima et al. [33]	700

## Data Availability

Not applicable.

## Abbreviations

MT	microtubule
MTD	microtubule doublet
CP	central pair
RS	radial spoke
ATP	adenosine triphosphate
IDA	inner dynein arm
ODA	outer dynein arm
DHC	dynein heavy chain
LC	light chain
IC	intermediate chain
CaM	calmodulin
MIA	modifier of IDA
CSC	spoke-associated complex
N-DRC	nexin-dynein regulatory complex
ER	endoplasmic reticulum
SR	sarcoplasmic reticulum
CICR	calcium induced calcium release
PKC	protein kinase C
IP_3_	inositol triphosphate
IP_3_R	inositol triphosphate receptor
CICI	calcium induced calcium influx
CTT	carboxyl terminal tail
